# Combinatorial analysis and algorithms for quasispecies reconstruction using next-generation sequencing

**DOI:** 10.1186/1471-2105-12-5

**Published:** 2011-01-05

**Authors:** Mattia CF Prosperi, Luciano Prosperi, Alessandro Bruselles, Isabella Abbate, Gabriella Rozera, Donatella Vincenti, Maria Carmela Solmone, Maria Rosaria Capobianchi, Giovanni Ulivi

**Affiliations:** 1 Clinic of Infectious Diseases, Catholic University of the Sacred Heart, Rome, Italy; 2 Department of Pathology, Immunology and Laboratory Medicine, Emerging Pathogens Institute, College of Medicine, University of Florida, Gainesville, Florida, USA; 3 Department of Virology, National Institute for Infectious Diseases "L. Spallanzani", Rome, Italy; 4 Department of Computer Science and Automation, faculty of Computer Science Engineering, University of Roma TRE, Rome, Italy

## Abstract

**Background:**

Next-generation sequencing (NGS) offers a unique opportunity for high-throughput genomics and has potential to replace Sanger sequencing in many fields, including de-novo sequencing, re-sequencing, meta-genomics, and characterisation of infectious pathogens, such as viral quasispecies. Although methodologies and software for whole genome assembly and genome variation analysis have been developed and refined for NGS data, reconstructing a viral quasispecies using NGS data remains a challenge. This application would be useful for analysing intra-host evolutionary pathways in relation to immune responses and antiretroviral therapy exposures. Here we introduce a set of formulae for the combinatorial analysis of a quasispecies, given a NGS re-sequencing experiment and an algorithm for quasispecies reconstruction. We require that sequenced fragments are aligned against a reference genome, and that the reference genome is partitioned into a set of sliding windows (amplicons). The reconstruction algorithm is based on combinations of multinomial distributions and is designed to minimise the reconstruction of false variants, called *in-silico *recombinants.

**Results:**

The reconstruction algorithm was applied to error-free simulated data and reconstructed a high percentage of true variants, even at a low genetic diversity, where the chance to obtain *in-silico *recombinants is high. Results on empirical NGS data from patients infected with hepatitis B virus, confirmed its ability to characterise different viral variants from distinct patients.

**Conclusions:**

The combinatorial analysis provided a description of the difficulty to reconstruct a quasispecies, given a determined amplicon partition and a measure of population diversity. The reconstruction algorithm showed good performance both considering simulated data and real data, even in presence of sequencing errors.

## Background

Next-generation sequencing (NGS) techniques [1-5] allow for a high-throughput DNA sequencing, producing from thousands to billions of sequence fragments (reads) composed of tens to hundreds of nucleotide bases. NGS has the potential to replace Sanger sequencing for many applications, including de-novo sequencing, re-sequencing, meta-genomics and intra-host characterisation of infectious pathogens [6-9].

De-novo sequencing implies a genome assembly problem, which is the reconstruction of a unique genome from a set of sequence fragments. Several methods and software for genome assembly have been developed [10-14]. These methods were designed initially for Sanger sequencing, and have been revised for NGS technology [15-18], given different error rates among NGS machineries [19,20]. Re-sequencing conjugates with the problem of single nucleotide polymorphisms (SNP) discovery. Recent studies characterised SNPs or drug-induced mutations with NGS, considering the human immunodeficiency virus (HIV) and the hepatitis B virus (HBV) [21,22]. More specifically, re-sequencing can be useful for the characterisation of variants within a quasispecies harbouring an infected host.

Many RNA viruses are present in a carrier (e.g. an infected patient) as a swarm of highly genetically related variants, i.e. a quasispecies, due to the error prone characteristics of the viral polymerases and high viral replication rates. This intra-host variability represents a substrate for the selective pressure exerted by the immune system of the host or by drug exposure, which leads to the continuous evolution of viruses. Quasispecies reconstruction would allow detailed description of the composition of individual viral genomes, genetic linkage and evolutionary history. For example, in HIV or HBV infection, the development of drug resistance is a major problem and the early diagnosis of drug-resistant variant selection might help in designing targeted therapeutic interventions.

Here, we addressed the problem of reconstructing a viral quasispecies from a NGS data set, which is a relatively new topic that is not widely investigated in literature. We aimed to reconstruct all coexistent individual variants within a population, along with their prevalence, rather than a reconstruction of a single or predominant genome. Current assembly software is not designed to accomplish this task, nor to deal easily with the reconstruction of highly variable genomes. The huge coverage and base pair output provided by NGS enables the design of experiments to investigate and validate theoretical methods for quasispecies reconstruction.

At present, only a few methodological papers have been published presenting new algorithms for quasispecies reconstruction that are able to infer both genomes of population variants and their prevalence [23-25]. In [23], the authors proposed an algorithm based on a generative model of the sequencing process and a tailored probabilistic inference and learning procedure for model fitting. In [24], a set of methodologies was proposed both for error correction and inference about the structure of a population from a set of short sequence reads as obtained from NGS. The authors assumed a known mapping of reads to a reference genome, defined a sliding window over the reference genome and associated each aligned read to one or more windows by trimming the reads accordingly to the windows' bounds. Sequencing errors were corrected by locally clustering reads in the windows. A set of single variants of the quasispecies (defined as haplotypes) was obtained by constructing an overlap graph of non-redundant, error-free, aligned reads, and by calculating a minimal coverage set of paths over the graph. The frequency estimation was done with an expectation maximisation algorithm and was proven to be more efficient than a naïve procedure based on uniform read sampling. One drawback of this methodology is that the variant reconstruction phase did not account for the relations among frequencies of distinct variants (counts of each distinct read representative) that were overlapping consistently across the sliding windows: this may lead potentially to selection of *in-silico *recombinants and the procedure of haplotype frequency may be biased from the exclusion of real (not selected) paths. After the paper, free software was released, named ShoRAH [26]. In [25], a scalable assembling method for quasispecies based on a novel network flow formulation was presented, applied efficiently for the assembly of Hepatitis C virus. In [27], a refinement of the original procedures presented in [24] was given, substituting k-means clustering with a Dirichlet process mixture for locally inferring haplotypes and correcting reads.

In this work, a set of formulae for combinatorial analysis of quasispecies genome fragments sampled by NGS was derived, and a new greedy algorithm for quasispecies reconstruction was introduced. The formulae derivation provided some theoretical bounds explaining the difficulty in reconstructing a set of individual variants of the quasispecies, by conditioning on several parameters, such as the genome length, the fragment (read) size, or the overlap length between two sampled fragments. The reconstruction algorithm was based on combinations of multinomial distributions and was designed to minimise the reconstruction of *in-silico *recombinants. Unlike previous approaches, the algorithm selects and reconstructs variants not only by coupling reads that have consistent overlaps, but also considering reads that have similar frequencies across the various amplicons.

For our combinatorial analysis, we assumed that the problem of re-sequencing, including reference alignment and error correction, is solved. In other words, a set of error-free reads is available, aligned univocally to a reference or consensus sequence. Such a reference may either have been directly reconstructed using assembly software or selected from literature. The assumption for the unique mapping of a read against the reference may not be always fulfilled when in presence of short reads and genomes with long repeats. However, this problem can be negligible when considering coding regions of highly-variable viral pathogens targeted by inhibitors, with a few regulatory regions where the repeats usually are located. The sequence quality may be a major concern for reconstruction algorithms and this is often an experiment- and machine-dependent problem: procedures for alignment and error corrections have been investigated elsewhere [16,17,24,27,21-30], with different methodologies, along with protocols for sample preparation. Another critical point with NGS is the presence of contaminants that must be detected and excluded. The problem can be solved easily when the contamination is from different organisms, with a test statistic on read/reference alignment scores during re-sequencing [31]. It is harder when the NGS experiment comprises a mixture of closely related organisms, for instance when samples of patients infected with the same virus are put together in one NGS experiment [29,30].

As a second assumption, our algorithm required a non empty set of overlapping regions, called *amplicons*, which cover the reference genome. Each read has to be assigned to one of these amplicons. Roche 454 GSFLX technology has a double working modality that allows both for shotgun sequencing and for amplicon sequencing with specific primer design, although the latter option is generally more expensive. With this technology, amplicons can be defined *a priori*. In contrast, if shotgun sequencing is performed, additional data elaboration has to be made in order to define a set of amplicons: one solution is to define amplicons via sliding windows over the genome and cluster reads accordingly to their mapping region [24].

The proposed reconstruction algorithm was applied to 1) simulated and error-free data; and 2) then empirical sequence data derived from blood samples from HBV-infected patients processed via the Roche 454 GSFLX Titanium machine. This second dataset was designed to assess the performance of quasispecies reconstruction in presence of sequencing errors.

## Results

### Algorithm: NGS data processing and amplicon definition

This work analysed a re-sequencing experiment of a viral quasispecies carried out using NGS machinery. Since currently the maximum read length of a NGS experiment does not exceed a few hundred of bases, we were interested in genome regions of quasispecies whose length is much larger than the average read length, i.e. when it is not possible that a read spans entirely the genome of interest. We required then that a reference genome is available and that reads are significantly aligned (mapped) against this reference genome. This can be achieved by aligning each read in forward- or reverse-strand against the reference genome, using the Smith-Waterman-Gotoh local alignment [32], which is an exact algorithm, and keeping the highest alignment score. Reads then can be filtered by excluding those that do not show a significant (for example, p < 0.01) alignment score, as compared to a score distribution obtained from quasi-random sequences (same average length, standard deviation and nucleotide content w.r.t. the original read set) aligned to the reference genome, as described in [31].

We assumed also that sequencing errors were corrected. The condition of error-free reads was required only for the combinatorial analysis, whilst the quasispecies reconstruction algorithm can be applied also to noisy data (as it was shown in the testing section, on a real data experiment).

**Figure 1:**
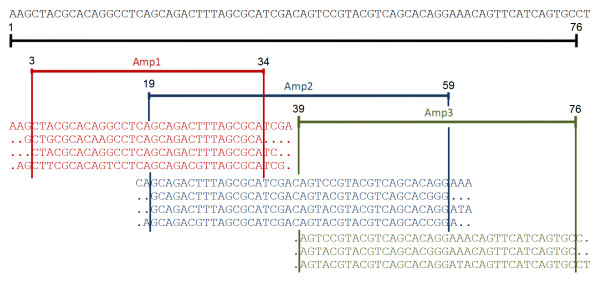
**Amplicon design**. Sliding-window amplicon design example for an hypothetical re-sequencing experiment over a NGS read sample aligned to a reference genome. Reads that cover entirely the amplicon window are retained and trimmed to fit the start/end amplicon positions.

Given a reference genome *g *and a read alignment over *g*, we define then a sliding window partition of *g *composed of *w+1 *windows, that we call *amplicons*. These amplicons cover the entire genome and two adjacent amplicons have a partial overlap, for a total of *w *overlaps. Amplicons do not need to be necessarily of the same length. Clearly, each amplicon size has to be smaller than the average read size, so that a read can span an amplicon entirely. As stated in the introduction, amplicons can be designed a priori if Roche 454 GSFLX technology is used, or determined with a fixed sliding window approach from any shotgun sequencing. After defining the amplicons, reads that spanned entirely an amplicon were trimmed so that their start/end positions corresponded exactly to the amplicon start/end. Consequently, all the reads in one amplicon had the same length. Reads that span more than one amplicon entirely are considered multiple times, whilst those that do not cover exactly at least one amplicon were discarded. Figure 1 illustrates with an example a three-amplicon design over a reference genome, with corresponding read assignment and trimming.

### Algorithm: combinatorial analysis of NGS over a quasispecies

Consider a number of *x *variants (that induce a quasispecies) determined by their genomic sequences of length *n*, either nucleotidic or amino-acid (or any other desired alphabetic coding scheme). The number *x *of variants is unknown, along with the prevalence of each variant.

We assume that the quasispecies is stable over a fixed set of variants, in a mutation-selection balance [33,34]. In other words, the number of distinct viral variants in the quasispecies that a carrier (e.g. an infected patient) harbours is *x*, although each variant can be present with different prevalence, presumably due to different viral fitness or immune response or drug-induced selection. After a multiple sequence alignment, a consensus sequence can be generated or one variant can be used as a reference. We define a *point difference *between two aligned sequences (or a *point mutation *from one sequence with respect to another) as the presence of two different nucleotides in one position of the alignment. The *pairwise difference *between two variants *d(s_i_, s_j_) *is the number of point differences divided by the genome length (*n*), i.e.

(1) $$d(s_{i},s_{j})=d_{ij}=\frac{\sum_{k=1}^{n}\left(s_{ik}\neq s_{jk}\right)}{n}$$

The average pairwise difference of a variant *s_i _*with respect to all the others is defined as

(2) $$d\left(s_{i}\right)=d_{i}=\left(\sum_{j\neq i}d_{ij}\right)/\left(x-1\right),\text{ for }j=1...x.$$

Finally, the average overall pairwise difference among a set of aligned variants is then

(3) $$d_{avg}=\frac{\sum_{i=1}^{x-1}\sum_{j=i+1}^{x}d_{ij}}{\frac{x(x-1)}{2}}$$

Let us define now a reference sequence *s_ref _*as the variant with the lowest average pairwise difference as compared to all other variants, i.e. *d_ref _= d(s_ref_) *= min{*d(s_i_)*}, for *i = 1...x*.

If we define the *diversity *- that we regard as a probability of mutation - of the quasispecies as *m = d_avg_*, then we can also approximate *m *by doubling the value of *d_ref_*, i.e. *m/2 *is the average pairwise difference of our reference variant with respect to any other variant (*m/2 = d_ref_*). Indeed, this approximation is correct when no identical base changes (mutations) happen in the same alignment position of two variants as compared to the reference, and this is dependent on the genome length and the mutation probability. The approximation gets better either if the genome length increases or the mutation rate decreases (by keeping one of the two values constant). More details on the efficacy of this approximation are given in Additional file 1.

We previously defined a joined set of *w+1 *amplicons, which induces an overlapping ordered coverage over the quasispecies genome space. Assume that each amplicon has a fixed length of *k *bases and overlaps with its neighbour(s) over *q *bases for *w *times. We assume also that, given three adjacent amplicons and two corresponding overlaps, these latter do not share any position in common, i.e. there are no overlapping overlaps. Since there are no nested amplicons, we can define an amplicon identification number as its ordinal position with respect to the reference genome and the other amplicons. Thus, *amplicon_1 _*starts at position *1 *and ends at position *k*, *amplicon_2 _*starts at *k-q+1 *and ends at *2k-q*, et cetera. The overlaps are clearly at the end of each amplicon and at the beginning of the adjacent one.

Each amplicon is associated with a set of reads, or sequence fragments, sampled uniformly from the quasispecies. These reads, by definition, are significantly aligned to the reference genome, error-free and span exactly an amplicon region, being trimmed at its ends. Thus, after sampling, each count of distinct reads across each amplicon cannot exceed the number of variants *x*.

Given two reads associated to two adjacent amplicons, we say that their overlapping region is *consistent *if the two reads share in that region the same characters (for instance over the alphabet {A, C, G, T} when considering nucleotide sequences).

We aim to calculate the probability that (i) the overlapping region of two adjacent reads is consistent and (ii) at least one overlapping region across the amplicons is consistent.

For point (i), we first define the probability that *i *mutations are present in a sequence fragment of *q *length over a genome of length *n *(that can be conceptually associated to one overlap) as

(4) $$p(i|q,n,m)=\frac{\left(\begin{array}{l} q\\ i \end{array}\right)\left(\begin{array}{l} n-q\\ nm/2-i \end{array}\right)}{\left(\begin{array}{l} n\\ nm/2 \end{array}\right)}$$

Note that the diversity *m/2 *here is multiplied by *n *(and assumed integer), obtaining the expected number of changes (*nm/2*).

The probability that two random regions of *q *length over a genome of length *n *have both *i *mutations is

(5) $$p(i_{1}=i_{2}=i|q,n,m)={\left(\frac{\left(\begin{array}{l} q\\ i \end{array}\right)\left(\begin{array}{l} n-q\\ nm/2-i \end{array}\right)}{\left(\begin{array}{l} n\\ nm/2 \end{array}\right)}\right)}^{2}$$

where the terms of Eq. 4 are the square root of the terms of Eq. 5.

The probability that these random regions share exactly *i *mutations at the same positions (regardless their positioning in the genome) is

(6) $$p(i_{1}=i_{2}=i\wedge (\text{pos}(i_{1})=\text{pos}(i_{2}))|q,n,m)=\frac{{\left(\frac{\left(\begin{array}{l} q\\ i \end{array}\right)\left(\begin{array}{l} n-q\\ nm/2-i \end{array}\right)}{\left(\begin{array}{l} n\\ nm/2 \end{array}\right)}\right)}^{2}}{\left(\begin{array}{l} q\\ i \end{array}\right)}\cdot {\left(1/3\right)}^{i}$$

where the term *(1/3)^i ^*accounts for the 4-letter alphabet since we are considering nucleotides. In the binary case, the term has to be dropped. In the general case, for an alphabet of size *σ*, it would correspond to *(1/(σ-1))^i^*.

Thus, the probability that the two sequence fragments are the same is

(7) $$p(fragment_{1}=fragment_{2}|q,n,m)=\sum_{i=0}^{i=nm/2}\left(\begin{array}{l} q\\ i \end{array}\right){\left(\frac{\left(\begin{array}{l} n-q\\ nm/2-i \end{array}\right)}{\left(\begin{array}{l} n\\ nm/2 \end{array}\right)}\right)}^{2}\cdot {\left(1/3\right)}^{i}$$

In our context, ***fragment**_1 _*and ***fragment**_2 _*refer to the overlapping region of two distinct reads in two adjacent amplicons.

For point (ii), let's define the set *A = {(a_1_, a_2_, ..., a_w_, a_w+1_) | a_i _∈ N, a_1_+a_2_+...+a_w_+a_w+1 _= nm/2}*, as the space of frequency distributions where *nm/2 *mutations can distribute either in *w *overlaps or in the remaining (non overlapping) parts, grouped in the additional variable *w+1*. Given a generic element ***a **= (a_1_, a_2_, ..., a_w_, a_w+1_) ∈ A*, each *a_i _*contains a certain number of mutations and the sum is the total number of mutations.

Of note, the formula that gives the number of elements of the space *A*, as a function of *n, m *and *w *is

(8) $$|A|=\left(\begin{array}{l} w+1-1+nm/2\\ nm/2 \end{array}\right)=\left(\begin{array}{l} w+nm/2\\ nm/2 \end{array}\right)$$

and corresponds to the number of combinations with repetitions of *w+1 *elements of *nm/2 *class.

The probability that *nm/2 *mutations distribute over the overlaps and the non-overlapping parts in a mode *(a_1_, a_2_, ..., a_w_, a_w+1_) *is

(9) $$p\left(\left(a_{1},\;a_{2},\;...,\;a_{w},\;a_{w+1}\right)|q,n,m,w\right)=\frac{\left(\begin{array}{l} q\\ a_{1} \end{array}\right)\left(\begin{array}{l} q\\ a_{2} \end{array}\right)...\left(\begin{array}{l} q\\ a_{w} \end{array}\right)\left(\begin{array}{l} n-qw\\ a_{w+1} \end{array}\right)}{\left(\begin{array}{l} n\\ nm/2 \end{array}\right)}$$

Thus, for two vectors ***a **= (a_1_, a_2_, ..., a_w+1_) ∈ A *and ***b **= (b_1_, b_2_, ..., b_w+1_) ∈ A*, at least one overlapping region (over the *w *set) will be consistent if, excluding the non-overlapping part, either (ii.1) both ***a ***and ***b ***have the same element set to zero (i.e. *∃ i | a_i _= b_i _= 0, i ≠ w+1*) or (ii.2) both have one or more identical elements in the same overlap and within this overlap the mutations are in the same sites (*∃ i | a_i _= b_i_, i ≠ w+1, a_i _≠ 0, **fragment**_ai _*= ***fragment**_bi_*).

For case (ii.1), let *p(**a_i_**) *be the probability (which can be calculated with Eq. 9) for a generic distribution ***a_i _**= (a_i1_, ..., a_ik_, ..., a_i(w+1)_) ∈ A*, where at least one element *a_ij _*is equal to zero. Define *p_ij _*as the joint probability between two distributions, i.e. *p_ij _= p(**a_i_**)p(**a_j_**). *The sum of all joint probabilities *∑p_ij_*, where *∀ i ∃ j, k | a_ik _= a_jk _= 0, k ≠ w+1*, yields the probability of a consistent overlap.

For case (ii.2) we show how to calculate the probability associated to two distributions ***a ***and ***b***, when they share at least one identical element, different from *0*, otherwise the case reduces to (ii.1).

Consider the two products

(10) $$\begin{array}{l} \pi_{1}=\left(\begin{array}{l} q\\ a_{1} \end{array}\right)\left(\begin{array}{l} q\\ a_{2} \end{array}\right)...\left(\begin{array}{l} q\\ a_{w} \end{array}\right)\left(\begin{array}{l} n-qw\\ a_{w+1} \end{array}\right)\\ \pi_{2}=\left(\begin{array}{l} q\\ b_{1} \end{array}\right)\left(\begin{array}{l} q\\ b_{2} \end{array}\right)...\left(\begin{array}{l} q\\ b_{w} \end{array}\right)\left(\begin{array}{l} n-qw\\ b_{w+1} \end{array}\right) \end{array}$$

and choose one of them, say *π_1_*.

If the two distributions have *j *identical elements (number of mutations) in the same sites (overlaps, from *1 *to *w*, and non-overlapping part), naming them *1, 2, ..., j*, we can write the following

(11) $$\begin{array}{l} \alpha_{1}=\pi_{1}-\frac{\pi_{1}}{\left(\begin{array}{l} q\\ a_{1} \end{array}\right)}\cdot {\left(1/3\right)}^{a_{1}}\\ \alpha_{2}=\alpha_{1}-\frac{\alpha_{1}}{\left(\begin{array}{l} q\\ a_{2} \end{array}\right)}\cdot {\left(1/3\right)}^{a_{2}}\\ \alpha_{j}=\alpha_{j-1}\frac{\alpha_{j-1}}{\left(\begin{array}{l} q\\ a_{j} \end{array}\right)}\cdot {\left(1/3\right)}^{a_{j}} \end{array}$$

Any *α_j _*can be interpreted as the number of combinations (at each *j*, i.e. by considering *j *overlaps) that do not present the same elements in the same positions.

Finally

(12) $$p\left(a_{i}=b_{j}|q,n,m,w\right)=\frac{\left(\pi_{1}-\alpha_{j}\right)\pi_{2}}{{\left(\begin{array}{l} n\\ nm/2 \end{array}\right)}^{2}}$$

is the probability for the two generic distributions ***a ***and ***b ***to have at least one identical overlap. Note that Eq. 12 is valid under the constraint *q > = nm/2 *and *(n-wq) > = nm/2*. The sum of all joint probabilities *∑ p _ij_*, where *∀ i ∃ j, k | a_ik _= b_jk_, k ≠ w+1, a_ik _≠ 0*, yields the probability of a consistent overlap.

Eq. 12 is computationally intensive: for a small value of *w *and *n *it is possible to calculate it exactly, but for larger values (i.e. real cases), it is preferable to rely on numerical simulations.

### Algorithm: Reconstruction of the quasispecies

From the definition in the above paragraph, we have a set of *x *variants *(v_1_, ..., v_x_) *over a quasispecies, with a genome length of *n *and a mutation probability *m*. Each variant has an associated prevalence *p(v_1_), p(v_2_), ..., p(v_x_)*, such that *p(v_1_)+p(v_2_)+...+p(v_x_) = 1*. By using NGS machinery, we are able to sample (e.g. to sequence) uniformly a large number of variant sequence fragments from the quasispecies population. Upon the definition of amplicons, we obtain *w+1 *population samples, each one of length *k*, where *(w+1)k > n *and an amplicon overlap of *q *sites.

Previous studies investigated the probability of covering all bases of a single genome by shotgun sampling [35] and the probability of covering all bases of different variants in a quasispecies [24,36]. Nowadays, NGS machineries are able to cover with high support a quasispecies of genomes of a few kilobases length.

We define the multinomial distribution *C_i _= (c_i1_, c_i2_, c_i3_, ..., c_ix_), i = 1 ... (w+1)*, where the generic element *c_ij _*contains the number of identical reads (that are referred to variant *j*) found in the amplicon *i*; thus, we have *w+1 *available distributions. Since *x *is unknown, we assume initially that *x *is the maximum number of distinct reads that can be found in one amplicon, and we order the *c_ik _*decreasingly, assuming that, given two distributions *C_i _*and *C_j_*, each *c_ik _*and *c_jk _*correspond to a sample from the same variant.

Based on the samples and the corresponding distributions, we aim to reconstruct the genomes associated to the unknown variants and their number, which eventually may be different from the initial value of *x*.

The objectives may be easier to reach if all the amplicons were designed such that the variants were different in all the overlaps, if the number of reads sequenced and covering the amplicons was sufficiently large and if the reads were completely error-free. The problem becomes more challenging in presence of ambiguous overlaps (i.e. different variants that are identical in one or more overlap), non-uniform or biased sampling, and uncorrected read errors. For the latter (real) scenario, we design a set of algorithms in order to reconstruct a consistent set of variants that explains the *C_i _*distributions.

**Figure 2:**
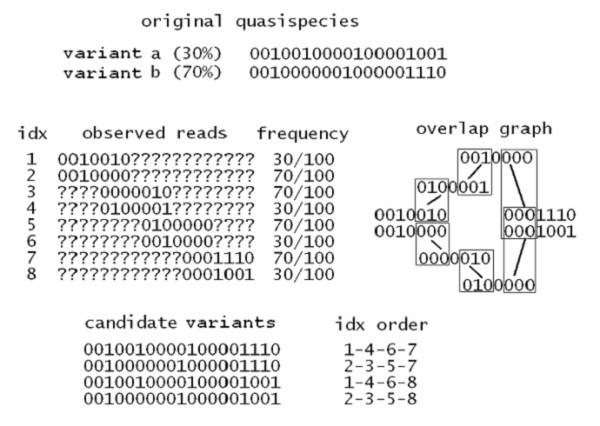
**Overlap graph**. Example of amplicon sampling from a quasispecies constituted by two variants (binary alphabet), with different prevalence. The reads are aligned to a reference genome, cover entirely an amplicon and are trimmed to the amplicon start/end positions (otherwise a question mark is placed). With such a design of 4 amplicons and 3 overlaps, the last overlap allows for ambiguous consistency. The overlap graph analysis leads to the reconstruction of 4 candidate variants, where 2 of them are *in-silico *recombinants. Without additional analysis on read distributions over the amplicons, it is impossible to infer the correct quasispecies.

Figure 2 shows one example assuming four amplicons (three overlaps) and two variants, over a binary alphabet. Assume an ideal sample from the population for each amplicon and error-free reads. From the figure, if we attempt to reconstruct the quasispecies based only on the graph of consistent overlaps, two *in-silico *recombinants would be constructed.

In the trivial case of a unique amplicon over the whole genome length, for a sufficiently large sample size, we may estimate variant probabilities from the distribution *C_1 _*as *p(v_i_) = c_1i_/∑_j_c_1j_*. With multiple amplicons, depending on *n, m, q*, *k *and sample size, the distributions *C_i _*vary: if the hypothesis of error-free reads was fulfilled, the equations of the previous paragraph permit to calculate some confidence bounds. In the real case, we expect that the multinomial distributions calculated for the amplicons are related, but we have to account for the uncertainty coming from the sampling process, cases of ambiguous overlaps and uncorrected read errors.

Having a set of *C_1_, ..., C_w+1 _*distributions, we may be interested to find which is the most probable distribution under a given set of parameters or a model, i.e. which distribution explains better the entire data. This would be useful when applying a reconstruction algorithm, as explained in the next paragraphs. If the probability of an event *X *dependent on parameter set ***θ ***(or model) is written *P(X | **θ**)*, then the likelihood of the parameters given the data is *L(**θ **| X)*. In our case, ***θ ***corresponds to one of the *C_i_*s and *X *is the set of remaining distributions *X = {C_j _| j = 1... w+1, j ≠ i}*. We aim to find *i *such that *L(C_i _| X) *is the maximum. However, since the derivation of *L(**θ **| X) *may be difficult, we use a minimum chi-square criterion [37]. For each *C_i_, i = 1... w+1*, calculate and sum the chi-square statistic associated with all other *C_j_*s, and pick up the index *i *for which the sum of chi-square statistics is the minimum. We may exclude as candidate model any *C_i _*for which *|C_i_| <*max*{ |C_j_| _j = 1...w+1 _}*.

We define now a procedure that reconstructs a set of candidate variants of the quasispecies: the procedure takes into account both read distributions over the amplicons and calculation of consistent overlaps. The algorithm is as follows:

1. Construct a matrix *M = (m_ij_), i = 1... x, j = 1... w+1*, where the columns represent the absolute frequency (i.e. counts) distributions of distinct reads in the amplicons and each row contains distinct read representatives with their associated frequencies. Thus, the generic element *m_ij _*is the number of distinct reads in amplicon *j *that correspond to a hypothetical variant *i*. Each column of the matrix is ordered decreasingly. Since *x *is estimated as the maximum number of distinct reads found considering each amplicon, in amplicons where the number of distinct reads is less than *x*, missing values are all set to *0*.

2. Choose a *guide *distribution among the amplicon distributions (either random or based on maximum likelihood), say the one corresponding to amplicon *g∈ {1, 2, ..., w+1}*.

3. For each *m_gj _∈ M, j = 1... x*, check iteratively if *m_gj _*is consistent with any other *m_ik_, i ≠ g, k = 1... x*. If there is more than one consistent overlap, choose the index *k *whose absolute difference with the actual *j *is the lowest (i.e. tend to join distinct reads according to their ordered prevalence).

3.1. When a consistent set of distinct reads is obtained, i.e. one variant is reconstructed with corresponding read-amplicon indices *{ĵ_1_, ..., ĵ_(w+1)_}*, subtract the number of distinct reads corresponding to the *m_gĵ _*value from the other *m_jĵ _*elements and update them in *M*. If some of the subtractions lead to negative values, set them to zero.

4. If there is not a column of *M *with all zero elements (*¬∃ j | ∀ i m_ij _= 0*) or if one variant has been constructed or the scan through amplicon distributions has not ended, go to point 2.

5. Output the variants reconstructed.

In the beginning, the algorithm counts all distinct reads for each amplicon. Distinct read representatives are ordered decreasingly by their frequency, creating *w+1 *multinomial distributions of size *x*, which is the maximum number of distinct reads seen considering each amplicon. If less than *x *distinct reads are found in one amplicon, the remaining elements of the corresponding multinomial distribution are set to zero-frequency. A guide multinomial distribution is chosen either at random or by the minimum chi-square criterion. The first read representative of the guide distribution corresponding to the count *m_g,1 _*is compared with the read representatives of an adjacent amplicon (say *g+1*), starting from the first read (at *m_g+1,1_*) checking if there is a consistent overlap between the two read representatives. If a consistent overlap is found, then a partial variant is reconstructed (now spanning 2 amplicons) and the step is repeated on another adjacent amplicon (*g+2 *or *g-1*), until the whole set of amplicons is analysed. If a consistent overlap between two reads is not found, for instance between read representatives corresponding to *m_g,1 _*and *m_g+1,1_*, then the procedure checks for a consistent overlap between the current read at position *m_g,1 _*and the next read in the same adjacent amplicon *g+1*, which is *m_g+1,2 _*in this case. Every time that a variant is reconstructed spanning all the amplicons, the algorithm subtracts the frequency count of the current read in the guide distribution (say *m_g,i_*) from the counts of all reads in the other amplicons that concurred to the variant reconstruction. Read frequencies that go to zero or below zero are considered exhausted and are not further evaluated. Negative values might appear due to variations generated by the NGS in the total read counts across the amplicons. Consider the trivial example of a quasispecies composed by a unique variant and two amplicons (with error-free sequencing), where in the first amplicon the total read count is 300 and in the second is 299. If the guide distribution corresponds to the second amplicon, the subtraction leads to -1. The algorithm stops when all reads have been examined or if one amplicon distribution has all zero-frequencies. A step-by-step example of the reconstruction algorithm is given in the Additional file 1. The computational complexity of the algorithm is *O(x^w^)*, which grows exponentially with the number of amplicons. Clearly, it is desirable to have a limited number of overlaps, e.g. of amplicons, in order to decrease the computational burden.

Note that initially the algorithm assumes that the number of variants in the quasispecies (which is unknown) is given by the maximum number of distinct reads observed across all amplicons (*x*), but the final number or reconstructed variants can be different, depending on the frequency distribution and overlap consistence. Indeed, in [24] it was shown that in some cases the number of variants is higher than the number of distinct reads. Using our algorithm, the number of variants would be exactly *x *if the multinomial distributions *m_i _*were allowing for just one consistent overlap between elements in the same row (i.e. variants only of the type *m_i1_, ..., m_ij_,..., m_i(w+1)_*) and if the frequency subtraction was always exhausting all the *m_ij_*.

In order to evaluate the effectiveness of the reconstruction algorithm and of the guide distribution choice policy, we designed and executed multiple simulation experiments over fixed parameters (*x, n, k, q*), varying mutation and sample size. Functions for the goodness of fit were (i) the prevalence of variants reconstructed correctly, (ii) the number of false *in-silico *recombinants, and (iii) number of reconstructed variants over the set of full consistent paths. We obtained distributions of these loss functions executing multiple simulation runs and compared them via parametric test statistics.

### Testing: combinatorial analysis

In the methods section we derived two main formulae that provide theoretical bounds for the probabilities of consistent overlaps. In particular, Eq. 7 describes the probability that one overlap is consistent, given the genome length, the number of amplicons and the overlapping region size.

Table 1 summarises these probabilities by varying the mutation rate, by setting *n *= 1,100, *q *= 50, *w+1 *= 7 (thus *k *= 200) over a genome described by a 4-letter alphabet (nucleotides). At a *m/2 *equal to 0.5%, for instance, the probability of a consistent overlap is 63%. At *m/2 *= 2.5%, the probability is still 8%.

**Table 1:** Probabilities to have a consistent overlap, given n = 1,100, q = 50, w+1 = 7, by varying the mutation probability m

*m/2 *(%)	Number of mutations in the overlap						total
		
	0	1	2	3	4	5	
0.5	6.27E-01	2.39E-04	2.85E-08	1.24E-12	1.44E-15	1.20E-20	6.28E-01

1	3.58E-01	6.67E-04	5.03E-07	2.00E-10	3.73E-12	1.54E-15	3.58E-01

1.5	2.23E-01	8.89E-04	1.52E-06	1.48E-09	7.37E-11	9.04E-14	2.24E-01

2	1.27E-01	9.64E-04	3.27E-06	6.57E-09	7.06E-10	1.97E-12	1.28E-01

2.5	7.86E-02	9.11E-04	4.79E-06	1.52E-08	2.63E-09	1.21E-11	7.95E-02

3.5	2.74E-02	6.42E-04	6.98E-06	4.69E-08	1.76E-08	1.81E-10	2.80E-02

More generally, Eq. 12 calculates the probability that at least one overlap is consistent. By fixing *n*, *q *and *w *as above, and by simulating Eq. 12 with 1 million of iterations, we calculated that the probabilities of at least one consistent overlap for *m/2 *= {0.5%, 1%, 1.5%, 2%, 2.5%, 3.5%} are *p *= {0.9992, 0.9460, 0.8018, 0.5720, 0.4003, 0.1584}, respectively. For instance, at an *m/2 *rate of 2.5% there is 40% of chance that at least one overlap is consistent. This gives a description of how much it could be difficult to reconstruct exact variants when the diversity is low.

### Testing: reconstruction algorithm on simulated data

The reconstruction algorithm, along with the investigation of guide distribution choice, was evaluated using simulated data. A quasispecies composed by *x *= 10 variants was designed, considering a genome of length *n *= 1,100 over a 4-letter alphabet. Variant prevalence was the following: *p(v_1_) *= 2%*; p(v_2_) *= 4%*; p(v_3_) *= 5%*; p(v_4_) *= 7%*; p(v_5_) *= 9%*; p(v_6_) *= 11%*; p(v_7_) *= 13%*; p(v_8_) *= 14%*; p(v_9_) *= 17%*; p(v_10_) *= 18%. The amplicons consisted of *w+1 *= 7 regions, each one of length *k *= 200 and overlap *q *= 50. Different uniform mutation probabilities were considered, specifically: *m/2 *= {0.5%, 1%, 1.5%, 2%, 2.5%, 3.5%}. We tested either a random guide distribution or a guide distribution chosen by maximum likelihood.

**Figure 3:**
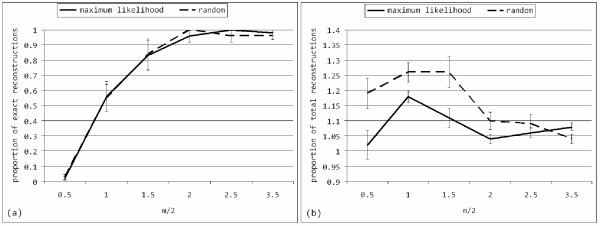
**Performance of the reconstruction algorithm (simulated data)**. Simulation results (average and standard error) for quasispecies reconstruction algorithm runs (*10*) for parameter set of *n = 1,100, w+1 = 7, k = 200, q = 50, x = 10*, sample size of *10,000*, varying *m *and guide distribution selection policy (continuous line for maximum likelihood, dashed for random choice). Panel (a) depicts proportion of correct reconstructions, while panel (b) proportion of total reconstructions.

We executed NGS simulations for a sample of 10,000 reads. The reads were error-free and uniformly distributed along the genome. Figure 3 reports simulation results over a set of 10 independent runs, shuffling the mutational sites.

With 10,000 read samples, the method reconstructed on average exactly the 10 variants at values of *m/2 *around 2%. By decreasing *m/2 *to 1%, on average more than a half of the original variants were reconstructed, but there was higher prevalence of *in-silico *recombinants. As it concerns the sole reconstruction of correct variants, comparison of the usage of a random guide distribution vs. one based on maximum likelihood did not yield significant differences. However, the maximum likelihood policy reconstructed, on average, a lower number of *in-silico *recombinants. Note that, since the multinomial distributions are ordered decreasingly, we expect to reconstruct variants from the most prevalent to the less prevalent.

**Figure 4:**
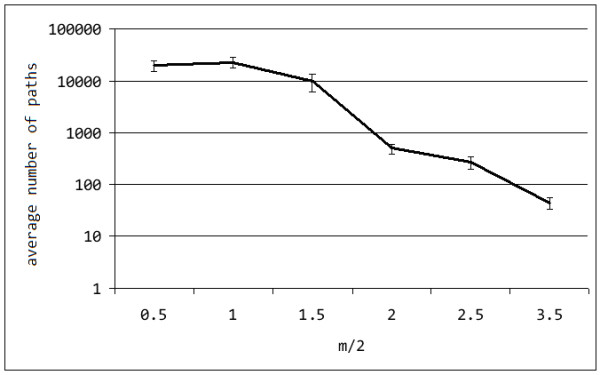
**Uncertainty in reconstructing variants**. Number of potential variants (over a true value of *x = 10*) by varying *m *(average per-site diversity), for parameter set of *n = 1100, w+1 = 7, k = 200, q = 50*, sample size of *10,000*, executing *10 *simulations.

Another way to evaluate the robustness of the algorithm is by looking at the number of potential variants (i.e. paths in the overlap graph) as a function of the per-site mutation probability, as depicted in figure 4.

In our simulation study, for an *m/2 *= 1% on average there would be ≈22,800 paths, i.e. candidate variants. Our algorithm on average chose 5-6 out of 10 correctly and did not reconstruct more than 10 variants. By increasing *m *to 1.5%, the number of paths would be still fairly high, i.e. ≈9,900: in this case the algorithm on average reconstructed > 80% variants correctly and the total number did not exceed 12.

**Figure 5:**
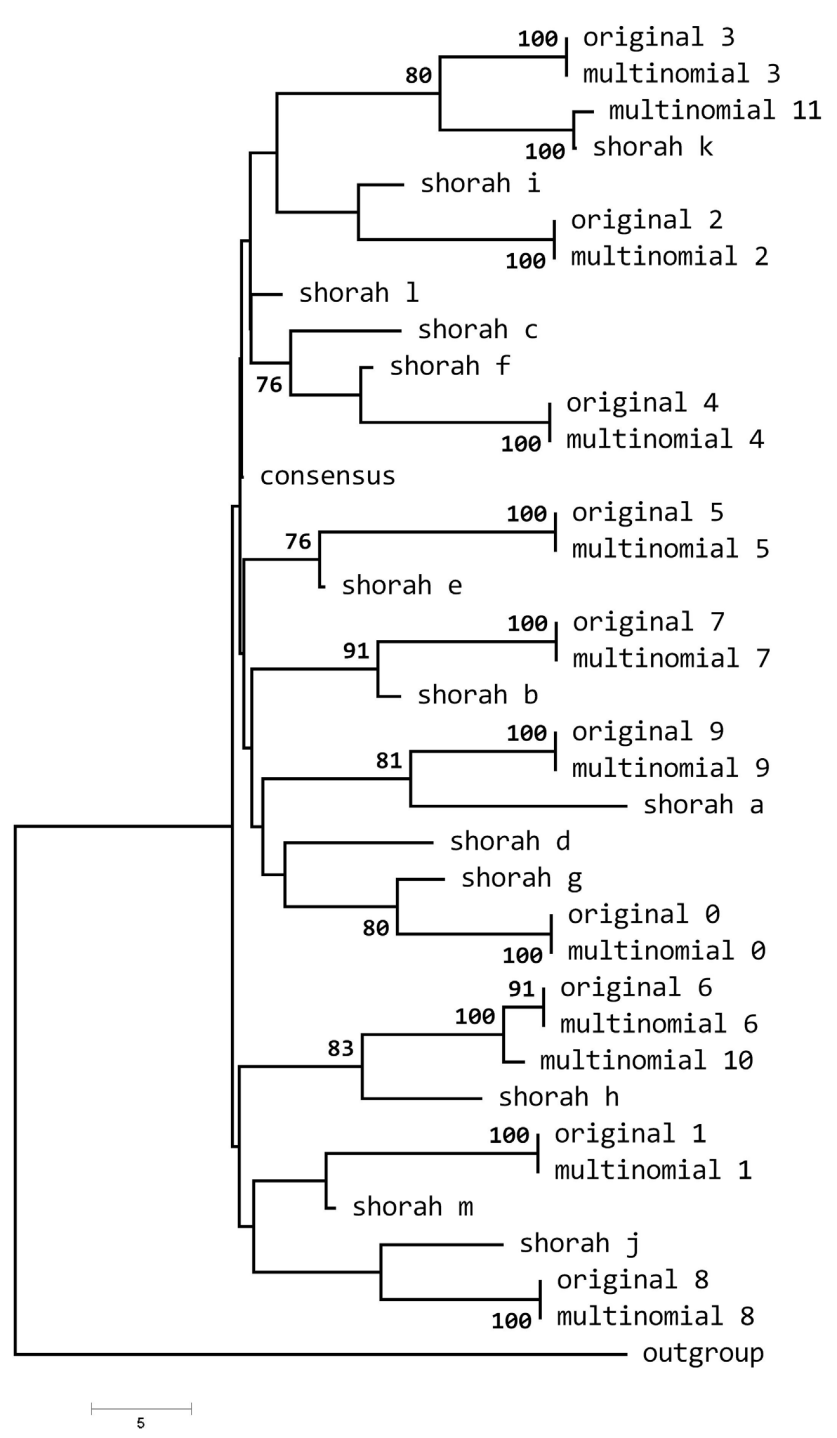
**Phylogeny of the reconstructed quasispecies (simulated data)**. Comparison between ShoRAH and our method on simulated data. A single simulation run is considered, consisting of *10,000 *reads sampled over a quasispecies of *10 *distinct variants (*m/2 = 1.5%, n = 1,100, q = 50, w+1 = 7*). The phylogenetic tree was constructed via Neighbor-Joining and distance based on simple number of differences, assessing branch significance through 100 bootstrap runs. Only nodes with a bootstrap support > 75% are indicated.

Using the same sets of simulated data (10 independent simulation runs with 10,000 read samples), we compared our algorithm with the ShoRAH (ver. 0.3.1, standard parameter set) program; however it should be noted that ShoRAH has not been designed to work on amplicons, but rather on shotgun modality. Although the current release provides the possibility to vary sliding window and the step size parameters, we could not reproduce exactly our amplicon settings, since the sliding window procedure is designed to cover multiple times each base over a uniform (i.e. shotgun) fragment sequencing. However, the average number of total reconstructions yielded by ShoRAH was comparable to our method, across different runs and *m *values. On average, at *m/2 *= 1.5%, the percentage of correct reconstruction was > 70% over different runs. Figure 5 depicts a phylogenetic tree constructed by pooling the original quasispecies together with the reconstructed variants from ShoRAH and our method, over a single simulation run at *m/2 *= 1.5%. Seven ShoRAH variants clustered significantly (> = 75% of node bootstrap support) with the original variants, over a total number of 13 reconstructions. Interestingly, our method reconstructed 12 variants (10 correct, 2 recombinants). A figure indicating recombination patterns is available in the Additional file 1.

### Testing: reconstruction algorithm on real data

The algorithm was also applied to real NGS data. We designed an experiment amplifying HBV sequences from 5 infected patient using a Roche 454 GSFLX Titanium machine based on the amplicon sequencing modality. Patients' samples were processed in the same plate using barcodes [29,30]. Three amplicons were defined with specific primers, each one with a length of {329, 384, 394} bases and with two overlaps of length {166, 109}. See the Additional file 1 for experiment details.

One patient was infected with a genotype A virus (12,408 reads) and four with a genotype D (5,874, 20,632, 4,900, and 6,598 reads, respectively). Overall, average (st.dev.) read length was 398.8 (71.1) bases.

The same HBV reference sequence (gi|22530871|gb|AY128092.1|) was used for read alignment and individual genome re-sequencing of each patient. We selected only reads that were significantly aligned with the reference (p < 0.01, using the Smith-Waterman-Gotoh local alignment with gap-open/extension penalties of 15/0.3 and the test statistic proposed in [31]). Three-percent of reads was discarded. The average diversity *m/2 *was 2.3%. According to the amplicon coverage, we reduced the amplicon lengths to {350, 350, 290} and overlaps to {150, 90} bases. Finally, we selected those reads that covered entirely one amplicon region with a gap percentage below 5%. For each amplicon, exactly 1,000 reads for patients were retained, selecting them at random, without replacement, from the previous set of filtered sequences. All reads from the different patients were pooled together in a unique file, thus obtaining 3,000 reads per patient and 15,000 reads in total, with a fixed read/amplicon/patient ratio. We were able to reconstruct virus consensus genomes from each individual using the read alignment, but we did not know a-priori the composition of the viral quasispecies of the patients. However, for each read we knew the corresponding patient. The purpose of this experiment was to see if the reconstruction algorithms were able to reconstruct a swarm of variants closely related to each patient's virus consensus genome, without mixing the population and without creating incorrect, populations.

**Figure 6:**
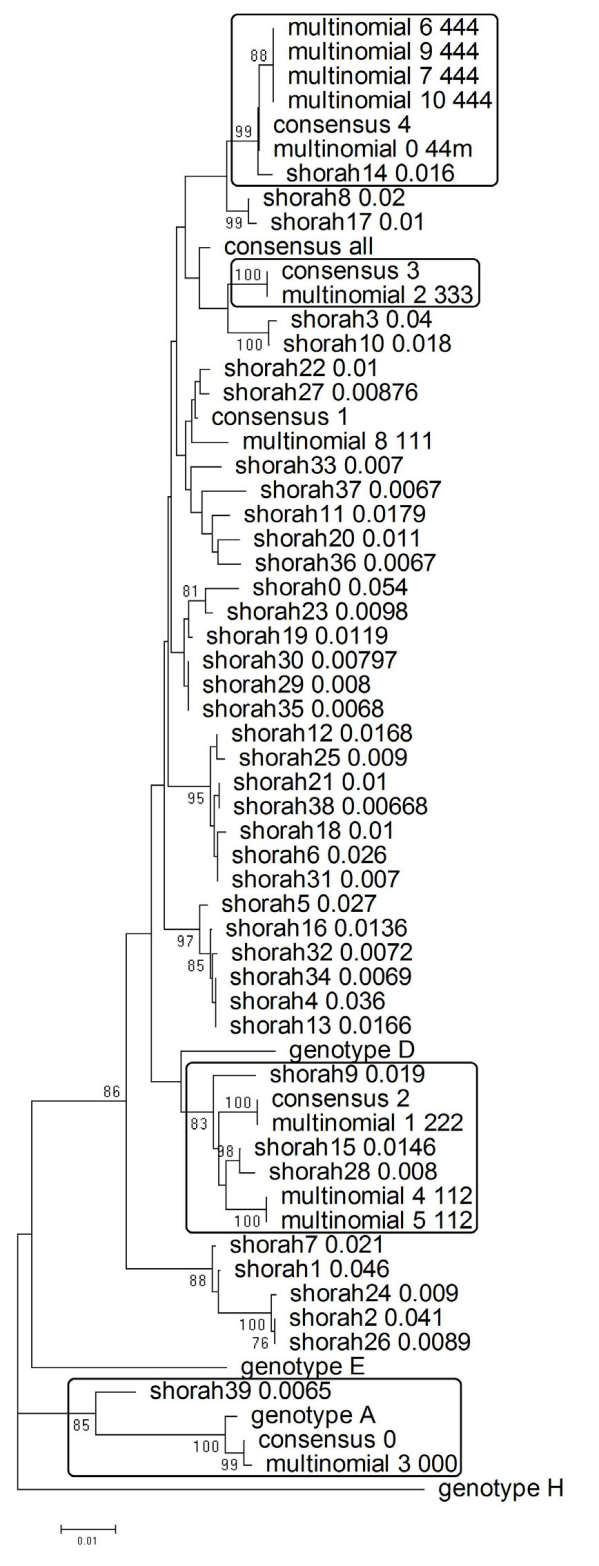
**Phylogeny of the reconstructed quasispecies (real data)**. Evaluation of reconstruction algorithms on real data (NGS experiment on 5 HBV-infected patients). The phylogenetic tree was constructed via Neighbor-Joining and LogDet distance, assessing branch significance through 1,000 bootstrap runs. Only nodes with a bootstrap support > 75% are indicated. Boxes comprise distinct patients' consensuses and reconstructed variants, when one or more reconstruction cluster significantly with them.

Both ShoRAH (ver. 0.3.1, standard parameter set) and the reconstruction algorithm were run on this joined data set, considering - as a simple error correction procedure - only reads with a frequency > = 3, requiring that at least one read was seen in reverse-strand and another in forward-strand. ShoRAH identified 854 distinct variants, with a median (IQR) prevalence of 0.00015 (0.00008-0.00038). The number of ShoRAH variants with prevalence above the 95^th ^percentile of the overall distribution was 40. Our reconstruction algorithm reconstructed 11 unique variants. We executed a phylogenetic analysis pooling together the set of reconstructed genomes, the 40 ShoRAH variants, the 11 unique variants obtained with our algorithms, and two additional outgroups (HBV genotypes H and E). The phylogenetic tree was estimated via a neighbour-joining method and the LogDet distance, assessing node support with 1,000 bootstrap runs. All the variants reconstructed with our algorithm clustered with the corresponding patients, and in four cases out of five the phylogenetic clusters had a support > 75%. The same held when looking at the ShoRAH variants, although a considerable number of variants clustered apart from the patients. Figure 6 depicts the phylogenetic tree. Of note, in patient #2, two variants reconstructed with our algorithm were indeed recombinants between patient #2 and patient #1.

## Discussion

In this paper we addressed the problem of quasispecies determination and variant reconstruction by using NGS machinery. Original assumptions were: (i) to have a uniform random sampling of the population, (ii) a reference genome, (iii) a unique, error-free, alignment of each read against the reference, and (iv) a sliding window partition of the reference into a set of amplicons. We derived first a set of formulae in order to analyse the probability of consistent overlaps given two sequence fragments over a set of amplicons. We showed that many factors, including diversity and overlap length, can affect the chance to detect spurious consistent overlaps. We introduced then the concept of multinomial distribution as a model for the classification of distinct reads and relative prevalence within amplicons. Upon this, we designed a greedy algorithm that reconstructs a set of paths through the whole set of amplicons (i.e. reconstructs candidate variants), coupling elements of different multinomial distributions, and trying to minimise the chance to reconstruct *in-silico *recombinants. The algorithm is based on a "guide distribution" policy that can be either random or based on maximum-likelihood. With a practical example (figure 2), we highlighted the reasons for which any quasispecies reconstruction procedure should consider read frequencies in order to avoid the estimation of false variants. In fact, our reconstruction algorithm tends to select variants not only looking at the consistent overlaps (e.g. reconstruction paths), but also considering reads that have similar frequencies across the various amplicons.

Simulation results proved that, exploring a fixed set of parameters, our method was able to select a compact and correct set of variants even at low diversities. At *m/2 *= 1.5%, the algorithm was able to reconstruct on average > 80% of correct variants, with an estimated number of variants close to the real value (12 over 10, where the total number of candidate variants was in the order of 10^4^).

We also executed a test on real NGS data, prone to contain sequencing errors, considering a mixed population of HBV-infected patients with a low average diversity. In this case our algorithm was able to distinguish variants corresponding to different patients, with a minimal evidence of *in-silico *recombination. In addition, the algorithm did not generate variants that could be different from the sequenced population.

In its current definition, though, our model possesses several limitations. First, a reference genome and a sliding window amplicon partition are needed: thus, the variant reconstruction method is suitable only for quasispecies for which there is at least one available genome. However, de-novo quasispecies determination can be easily achieved by pre-processing NGS data with existing whole genome assembly software and obtaining a usable reference genome.

Another important critical point is that we assume a uniform distribution of diversity along the genome, which is an ideal hypothesis. One solution may be the design of amplicons and overlaps of different lengths. The reconstruction algorithm works even with size-variable amplicons and overlaps, but the formulae introduced in the preliminary combinatorial analysis should be derived again, taking into account these modifications.

Another issue is the assumption of a unique mapping of each read with respect to the reference genome, which may be not always fulfilled when in presence of long repeats (compared to the average read length). However, this problem does not affect the reconstruction algorithm once the read mapping is given along with the sliding window amplicon setup. Several approaches have been proposed in literature [38] and may be applied to NGS.

As future refinements of the reconstruction algorithm we foresee the estimation of exact variant prevalence, since currently we report variants just in decreasing prevalence order: one idea is to calculate average and standard errors of distinct read frequencies from the various multinomial distributions joined during the reconstruction phase; another approach could be to estimate the prevalence a-posteriori, using expectation-maximisation as it was done in [24]. A broader perspective would be to relax the need for a reference genome and to estimate the quasispecies independently from the read mapping and the amplicon definition, but under these general settings the theoretical results here obtained would be hardly reusable.

## Conclusions

The presented combinatorial analysis and the reconstruction algorithm may be a fundamental step towards the characterisation of quasispecies using NGS. Immediate applications can be found in analysing genomes of infectious pathogens, like viruses, currently targeted by inhibitors and developing resistance. The investigation of in-depth, intra-host, viral resistance evolutionary mechanisms and interactions among mutations is crucial in order to design effective treatment strategies, even at early disease stages, and to maximise further treatment options.

## Authors' contributions

All authors read and approved the final manuscript. LP carried out combinatorial analysis and algorithmic specifications. MP performed simulations, evaluation on real data, phylogenetic analysis, statistical comparisons with other methods, and manuscript writing. GU revised the mathematical methods. AB provided software installations and runs. IA, GR, DV, and MCS provided expertise in next-generation machinery and amplicon sequencing, and performed laboratory experiments. MRC leaded the research and supervised authors' contributions.

## Supplementary Material

Additional file 1 **This file includes additional details on: (i) average population diversity estimation; (ii) step-by-step example of the quasispecies reconstruction algorithm; (iii) information on the sample preparation and laboratory protocols for the experiment on Roche GLSFLX platform; (iv) figure of recombination patterns for a reconstructed quasispecies given a simulation experiment**. Click here for file

## References

[B1] Roche 454 GSFLX http://www.454.com/

[B2] Illumina http://www.illumina.com/

[B3] SOLiD http://www3.appliedbiosystems.com/AB_Home/applicationstechnologies/SOLiDSystemSequencing/index.htm

[B4] Helicos http://www.helicosbio.com/

[B5] The Polonator http://www.polonator.org/

[B6] Wheeler DA Srinivasan M Egholm M Shen Y Chen L McGuire A He W Chen YJ Makhijani V Rothberg JM The complete genome of an individual by massively parallel DNA sequencing Nature 2008 452 872 876 10.1038/nature06884 18421352

[B7] Mardis ER The impact of next-generation sequencing technology on genetics Trends Genet 2008 24 3 133 41 18262675 10.1016/j.tig.2007.12.007 PMC2680276

[B8] Voelkerding KV Dames SA Durtschi JD Next-generation sequencing: from basic research to diagnostics Clin Chem 2009 55 4 641 58 10.1373/clinchem.2008.112789 19246620

[B9] Metzker ML Sequencing technologies - the next generation Nat Rev Genet 2010 11 1 31 46 10.1038/nrg2626 19997069

[B10] Bonfield JK Smith K Staden R A new DNA sequence assembly program Nucleic Acids Res 1995 23 24 4992 9 10.1093/nar/23.24.4992 8559656 PMC307504

[B11] Huang X Madan A CAP3: A DNA sequence assembly program Genome Research 1999 9 868 877 10.1101/gr.9.9.868 10508846 PMC310812

[B12] Myers EW Sutton GG Delcher AL Dew IM Fasulo DP Flanigan MJ Kravitz SA Mobarry CM Reinert KH Venter JC A whole-genome assembly of Drosophila Science 2000 287 5461 2196 204 10.1126/science.287.5461.2196 10731133

[B13] Batzoglou S Jaffe DB Stanley K Butler J Gnerre S Mauceli E Berger B Mesirov JP Lander ES ARACHNE: a whole-genome shotgun assembler Genome Research 2002 12 177 189 10.1101/gr.208902 11779843 PMC155255

[B14] Tammi MT Arner E Andersson B TRAP: Tandem Repeat Assembly Program produces improved shotgun assemblies of repetitive sequences Computational Methods Programs Biomed 2003 70 1 47 59 10.1016/S0169-2607(01)00194-8 12468126

[B15] Dohm JC Lottaz C Borodina T Himmelbauer H SHARCGS, a fast and highly accurate short-read assembly algorithm for de novo genomic sequencing Genome Research 2007 17 11 1697 706 10.1101/gr.6435207 17908823 PMC2045152

[B16] Li H Ruan J Durbin R Mapping short DNA sequencing reads and calling variants using mapping quality scores Genome Research 2008 18 1851 1858 10.1101/gr.078212.108 18714091 PMC2577856

[B17] Smith DR Quinlan AR Peckham HE Makowsky K Tao W Woolf B Shen L Donahue WF Tusneem N Richardson PM Rapid whole-genome mutational profiling using next-generation sequencing technologies Genome Research 2008 18 1638 1642 10.1101/gr.077776.108 18775913 PMC2556265

[B18] Miller JR Delcher AL Koren S Venter E Walenz BP Brownley A Johnson J Li K Mobarry C Sutton G Aggressive assembly of pyrosequencing reads with mates Bioinformatics 2008 24 24 2818 24 10.1093/bioinformatics/btn548 18952627 PMC2639302

[B19] Huse SM Huber JA Morrison HG Sogin ML Welch DM Accuracy and quality of massively parallel DNA pyrosequencing Genome Biol 2007 8 7 R143 10.1186/gb-2007-8-7-r143 17659080 PMC2323236

[B20] Philippe N Boureux A Bréhélin L Tarhio J Commes T Rivals E Using reads to annotate the genome: influence of length, background distribution, and sequence errors on prediction capacity Nucleic Acids Res 2009 37 15 e104 10.1093/nar/gkp492 19531739 PMC2731892

[B21] Wang C Mitsuya Y Gharizadeh B Ronaghi M Shafer RW Characterization of mutation spectra with ultra-deep pyrosequencing: application to HIV-1 drug resistance Genome Research 2007 17 8 1195 201 10.1101/gr.6468307 17600086 PMC1933516

[B22] Solmone M Vincenti D Prosperi MC Bruselles A Ippolito G Capobianchi MR Use of massively parallel ultradeep pyrosequencing to characterize the genetic diversity of hepatitis B virus in drug-resistant and drug-naive patients and to detect minor variants in reverse transcriptase and hepatitis B s antigen J Virol 2009 83 4 1718 26 10.1128/JVI.02011-08 19073746 PMC2643754

[B23] Jojic V Hertz T Jojic N Population sequencing using short reads: HIV as a case study Pacific Symposium on Biocomputing 2008 13 114 125 18229680

[B24] Eriksson N Pachter L Mitsuya Y Rhee SY Wang C Gharizadeh B Ronaghi M Shafer RW Beerenwinkel N Viral population estimation using pyrosequencing PLoS Comput Biol 2008 4 4 e1000074 10.1371/journal.pcbi.1000074 18437230 PMC2323617

[B25] Wesbrooks K Astrovskaya I Rendon DC Khudyakov Y Berman P Zelikovsky A HCV Quasispecies Assembly using Network Flows Proc. of International Symposium on Bioinformatics Research & Applications, LNBI 2008 4983 Springer Berlin/Heidelberg 159 170

[B26] ShoRAH http://www.bsse.ethz.ch/cbg/software/shorah

[B27] Zagordi O Geyrhofer L Roth V Beerenwinkel N Deep sequencing of a genetically heterogeneous sample: local variant reconstruction and read error correction LNCS 2009 5541 Springer Berlin/Heidelberg 345 358 10.1089/cmb.2009.0164 20377454

[B28] Campbell PJ Pleasance ED Stephens PJ Dicks E Rance R Goodhead I Follows GA Green AR Futreal PA Stratton MR Subclonal phylogenetic structures in cancer revealed by ultra-deep sequencing Proc Natl Acad Sci USA 2008 105 35 13081 6 10.1073/pnas.0801523105 18723673 PMC2529122

[B29] Hamady M Walker JJ Harris JK Gold NJ Knight R Error correcting barcoded primers for pyrosequencing hundreds of samples in multiplex Nature Methods 2008 5 3 235 237 10.1038/nmeth.1184 18264105 PMC3439997

[B30] Parameswaran P Jalili R Tao L Shokralla S Gharizadeh B Ronaghi M Fire AZ A pyrosequencing-tailored nucleotide barcode design unveils opportunities for large-scale sample multiplexing Nucleic Acids Res 2007 35 19 e130 10.1093/nar/gkm760 17932070 PMC2095802

[B31] Bacro JN Comet JP Sequence alignment: an approximation law for the Z-value with applications to databank scanning Computers and Chemistry 2000 25 401 410 10.1016/S0097-8485(01)00074-2 11459354

[B32] Gotoh O An improved algorithm for matching biological sequences J Mol Biol 1982 162 705 708 10.1016/0022-2836(82)90398-9 7166760

[B33] Eigen M McCaskill J Schuster P The molecular quasi-species Adv Chem Phys 1989 75 149 263 full_text

[B34] Domingo E Holland JJ RNA virus mutations and fitness for survival Annu Rev Microbiol 1997 51 151 178 10.1146/annurev.micro.51.1.151 9343347

[B35] Lander ES Waterman MS Genomic mapping by fingerprinting random clones: a mathematical analysis Genomics 1988 2 231 239 10.1016/0888-7543(88)90007-9 3294162

[B36] Chen K Pachter L Bioinformatics for whole-genome shotgun sequencing of microbial communities PLoS Comput Biol 2005 1 e24 10.1371/journal.pcbi.0010024 PMC1185649 16110337

[B37] Berkson J Minimum Chi-Square, not Maximum Likelihood! Ann Statist 1980 8 3 457 487 10.1214/aos/1176345003

[B38] Kececioglu JD Myers EW Combinatorial algorithms for DNA sequence assembly Algorithmica 1999 13 1 7 51 10.1007/BF01188580

