# Supplementary material for: Combinatorial analysis and algorithms for quasispecies reconstruction using next-generation sequencing
Source: BMC Bioinformatics. 2011 Jan 5;12:5. doi: 10.1186/1471-2105-12-5 (PMC3022557; doi:10.1186/1471-2105-12-5)

**Supplementary Material**

*Average population diversity*

In the main text we have considered as a reference sequence the variant with the lowest average pairwise difference, as compared to all other variants. We defined *d_ref_* as the average diversity obtained by comparing each sequence to that reference. The population diversity *m* (or *d_avg_*) was instead defined as the average diversity among all sequences. We said that, if the mutation probability is uniform over the genome, *d_ref_* is an approximation of *m/2*, in the particular case in which mutations do not appear in the same sites. We discuss here the more generic case in which one or more mutations can appear in the same sites.

Let us define two vectors s = (s_1_, s_2_, …, s_m/2_) and t = (t_1_, t_2_, …, t_m/2_) that represent the positions of the point mutations in two generic variants v_s_ and v_t_. The hypothesis that two point mutations do not appear in the same positions means that s_i_ is different from t_j_ for each i and j.

Note that the probability that two variants share j point mutations in the same position is

$$\frac{\left(\begin{matrix} m/2 \\ j \end{matrix}\right)\left(\begin{matrix} n-m/2 \\ m/2-j \end{matrix}\right)}{\left(\begin{matrix} n \\ m/2 \end{matrix}\right)}$$

We can calculate the probability that the j mutations do not appear in the same positions by fixing j=0. For instance, for a genome of length n=1,100 bases, at *m/2*=1, this probability is 89.5%

*Step-by-step example of quasispecies reconstruction algorithm*

Let’s construct the matrix *M* where each column represents a multinomial distribution of distinct reads for each amplicon. The multinomial distributions are all ordered decreasingly, as –for instance- in the following table (generated by 1,000 read samples)

| **legend** | | **amplicon** | | | | | | |
| --- | --- | --- | --- | --- | --- | --- | --- | --- |
|  |  | **1** | **2** | **3** | **4** | **5** | **6** | **7** |
| **Distinct reads** | **a** | 773 | 597 | 312 | 188 | 185 | 355 | 175 |
|  | **b** | 114 | 150 | 191 | 167 | 183 | 185 | 173 |
|  | **c** | 67 | 132 | 159 | 147 | 157 | 146 | 141 |
|  | **d** | 46 | 68 | 144 | 129 | 154 | 125 | 116 |
|  | **e** | 0 | 53 | 114 | 123 | 127 | 80 | 115 |
|  | **f** | 0 | 0 | 80 | 84 | 84 | 51 | 95 |
|  | **g** | 0 | 0 | 0 | 65 | 47 | 42 | 79 |
|  | **h** | 0 | 0 | 0 | 53 | 38 | 16 | 44 |
|  | **i** | 0 | 0 | 0 | 44 | 25 | 0 | 43 |
|  | **j** | 0 | 0 | 0 | 0 | 0 | 0 | 19 |

In this example amplicon no. 7 has 10 distinct reads with frequencies {175, 173, 141, 116, 115, 95, 79, 44, 19}. This may signify that (in an ideal case) there are exactly 10 variants in the quasispecies. Note that in the table zero-frequencies are assumed where the number of distinct reads in one amplicon is below the maximum. We choose now a guide distribution (say, the one corresponding to amplicon no. 7). From this guide distribution we try to reconstruct a variant by starting from the most frequent read (7.a, n=175) and checking if there is a consistent overlap among the other most frequent reads of each amplicon, i.e. 6.a, 5.a, 4.a, 3.a, 2.a, 1.a (n=355, 185, 188, 312, 597, 773). If, among this first set of reads, there is one non-consistent overlap (say, with 2.a) we pass to the next read (which is 2.b). Suppose that we get all consistent overlaps for the read sets

(773) of amplicon no. 1 (first read, 1.a)

(132) of amplicon no. 2 (third read, 2.c)

(191) of amplicon no. 3 (second read, 3.b)

(188) of amplicon no. 4 (first read, 4.a)

(183) of amplicon no. 5 (second read, 5.b)

(355) of amplicon no. 6 (first read, 6.a)

(175) of amplicon no. 7 (first read, 7.a)

Every time that a virus is reconstructed, we subtract the number of reads of the guide distribution from the other reads that were selected (i.e. had consistent overlap). Since the guide distribution is from amplicon no. 7, we subtract 175 from each one of the selected reads that become

(598) of amplicon no. 1 (1.a)

(-43) of amplicon no. 2 (2.c)

(16) of amplicon no. 3 (3.b)

(13) of amplicon no. 4 (4.a)

(8) of amplicon no. 5 (5.b)

(180) of amplicon no. 6 (6.a)

(0) of amplicon no. 7 (7.a)

Now the matrix has to be updated and distributions have to be re-sorted, such that

| **legend** | | **amplicon** | | | | | | |
| --- | --- | --- | --- | --- | --- | --- | --- | --- |
|  |  | **1** | **2** | **3** | **4** | **5** | **6** | **7** |
| **Distinct reads** | **a’** | 598 | 597 | 312 | 167 | 185 | 185 | 173 |
|  | **b’** | 114 | 150 | 159 | 147 | 157 | 180 | 141 |
|  | **c’** | 67 | 68 | 144 | 129 | 154 | 146 | 116 |
|  | **d’** | 46 | 53 | 114 | 123 | 127 | 125 | 115 |
|  | **e’** | 0 | 0 | 80 | 84 | 84 | 80 | 95 |
|  | **f’** | 0 | 0 | 16 | 65 | 47 | 51 | 79 |
|  | **g’** | 0 | 0 | 0 | 53 | 38 | 42 | 44 |
|  | **h’** | 0 | 0 | 0 | 44 | 25 | 16 | 43 |
|  | **i’** | 0 | 0 | 0 | 13 | 8 | 0 | 19 |
|  | **j’** | 0 | -43 | 0 | 0 | 0 | 0 | 0 |

Again, a new guide distribution must be chosen and the whole procedure has to be repeated. Every time that the distribution of distinct reads in one amplicon is exhausted without finding any consistent overlap, an adjacent amplicon is chosen and the next distinct read is evaluated. Note that, if it happens, negative frequencies of reads will be forced to zero. The whole procedure will stop ultimately when the last read from the guide distribution has been evaluated, or when a distribution has all zero-frequencies.

*NGS experiment on real data*

We used the 454 Life technology (Roche GS FLX platform, Titanium version) in order to detect and quantify viral variants and SNPs in the reverse transcriptase of HBV present in the serum of patients, by using the ultra-deep pyrosequencing approach, i.e. by using PCR to amplify overlapping segments of the region of interest, then the GS FLX to sequence the amplicons.

Since this technique is not able to sequence target fragments longer than 400 base pairs, primers were designed to amplify 3 partially overlapping segments, covering from aminoacid 48 to 324 of the reverse transcriptase gene. Three sets of primers were used, detailed in Table S1. In addition, the primers included 5’ extensions (left CGTATCGCCTCCCTCGCGCCA, and right CTATGCGCCTTGCCAGCCCGC adaptors, not shown in the table) which provided binding sites for the pyrosequencing on the genome sequencer. Furthermore a 10-bases barcode MID sequence, unique for each patient, was included between the primer and the adaptor sequence which permits to identify multiple samples pooled together.

HBV DNA was extracted using the QIAamp DNA Blood Mini Kit (Valencia, CA). All samples were amplified using a proof-reading enzyme (Fast Start High Fidelity enzyme, Roche, Mannheim, Germany). The conditions used for PCR were: 1 cycle of 95°C for 2 min followed by 40 cycles with denaturation for 30 sec at 95°C, annealing of primers for 30 sec at 58°C , and extension for 60 sec at 72°C with an additional 7 min for extension.

**Table S1.** Sequence and nt position of primers used for pyrosequencing of HBV

| HBV 1 FW | GCTGCTATGCCTCATCTTCT | 118-142 |
| --- | --- | --- |
| HBV 1 RW | GAYGATGGGATGGGAATAC | 471-489 |
| HBV 2 FW | TTGTTCAGTGGTTCGTMGGG | 289-305 |
| HBV 2 RW | GGGTTAAATGTATACCCAVAG | 689-710 |
| HBV 3 FW | TCTAGACTCGTGGTGGACTTCTC | 561-580 |
| HBV 3 RW | TACACAGAAAGGCCTTGTAAGTTG | 974-997 |

**Figure S1.** Comparison between ShoRAH and our method, over a single simulation run, consisting of *10,000* reads sampled over a quasispecies of *10* distinct variants (*m/2=1.5%, n=1,100, q=50, w+1=7*). Phylogenetic network constructed via Neighbor-Net and distance based on simple number of differences, assessing branch significance through 1000 bootstrap runs. Our method reconstructed a total of 12 variants, with 10 exactly matching the original ones. The 2 exceeding variants from our method were indeed in-silico recombinants of maximum two original species. Specifically, “multinomial 11” mixed original species 2 and 3 (half/half), while “multinomial 10” was composed by 83% of variant 6 and 17% of variant 2. Instead, ShoRAH was prone to show more complex patterns of recombination: the variant shorah_d was a recombinant of original species 0 and 9; shorah_a of original species 7 and 9; shorah_j of original species 1 and 8; shorah_h of original species 6 and 8; shorah_k of original species 2 and 3.


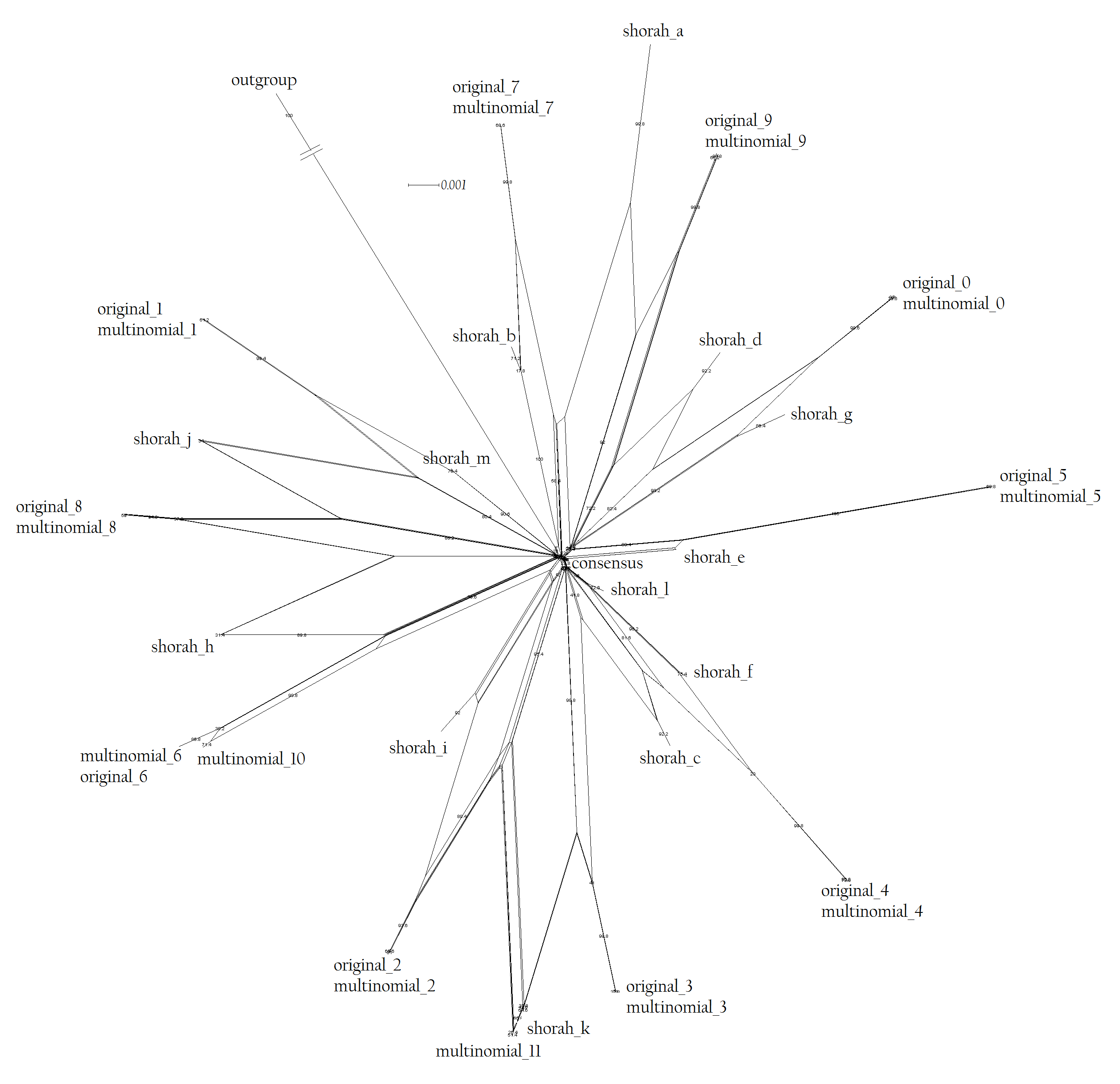

Supplement: Additional file 1 — This file includes additional details on: (i) average population diversity estimation; (ii) step-by-step example of the quasispecies reconstruction algorithm; (iii) information on the sample preparation and laboratory protocols for the experiment on Roche GLSFLX platform; (iv) figure of recombination patterns for a reconstructed quasispecies given a simulation experiment. [file 1471-2105-12-5-S1.DOC]
